# Methodology and experimental data of geometry-dependent temperature-time-profiles in laser-based powder bed fusion of polyamide 12

**DOI:** 10.1016/j.dib.2025.112293

**Published:** 2025-11-20

**Authors:** Joseph Hofmann, Benedikt Burchard, Valentin Krieger, Katrin Wudy

**Affiliations:** Technical University of Munich, TUM School of Engineering & Design, Department of Mechanical Engineering, Professorship of Laser-based Additive Manufacturing, 85748 Garching, Germany

**Keywords:** Additive manufacturing, Process monitoring, Infrared thermography, Polymer, Laser sintering

## Abstract

In laser-based powder bed fusion of plastics, the thermal history of the melt is essential to achieving high part properties. While the impact of part size on thermal behavior and properties is well understood, industrial processes frequently use fixed parameter sets, resulting in geometry-dependent variations in density and dimensional accuracy. This dataset documents the relationship between process parameters, thermal signals, and part properties resulting from laser processing. Experiments were carried out on an industrial system using polyamide 12 powder. A series of single-layer, cuboid, and tensile test samples with systematically varied energy input, scan speed, hatch distance, and scan vector length were produced under constant laser power. An infrared thermography camera monitored the temperature–time profiles during processing at 200 Hz. Characteristic features such as the peak of the thermal signal and decay time were extracted using automated data processing routines from these thermal signals. Complementary measurements include single-layer thicknesses, cuboid densities, dimensional accuracy measurements, and tensile testing. The thermal data are provided as raw, uncalibrated measurements, processed mean values, and standard deviations of extracted features for each sample. All data are openly available in standardized formats to facilitate reuse. Potential applications include predictive model development, benchmarking in situ monitoring methods, analyzing geometry-dependent parameter effects, and training adaptive control algorithms. A first analysis of this dataset is presented in a co-publication [1].

Specifications Table

**Table:** 

Subject	Engineering & Materials science
Specific subject area	Geometry-dependent thermal signals and part property data in laser-based powder bed fusion of polyamide 12
Type of data	Raw data: 1) NumPy files (.npy) of raw uncalibrated IR Thermography recordings (1 File per layer, top 6 layers of each sample); 2) Table with information to build job layout; Analyzed data: 3) Table with the process parameters, mean characteristic thermal features, and part properties (densities, dimensions, etc.) for each sample; 4) Table with mechanical properties (Tensile strength, elongation at break, stress at break, and Young’s modulus) 5) Figures showing 2D feature maps for exemplary thermal signals in the last layer of each sample 6) Figures showing diagrams that visualize the relationship between features of the thermal signal and part properties.
Data collection	Thermal data were collected with a mid-wave infrared thermography camera (200 Hz, 75 µm/pixel) from specimens fabricated using Polyamide 12 on an industrial PBF-LB/P system. Single-layer and cuboid samples were built using a 1:1 blend of virgin and used powder under systematically varied process parameters. Sample dimensions were measured with micrometer tools, and densities were determined via gravimetric and Archimedean methods. Furthermore, tensile samples are manufactured and tested. Thermal data were processed into NumPy arrays for analysis.
Data source location	Institution: Technical University of Munich, TUM School of Engineering & Design, Department of Mechanical Engineering, Professorship of Laser-based Additive Manufacturing; City/Town/Region: Garching by Munich; Country: Germany.
Data accessibility	Repository name: MediaTUM Data identification number: 10.14459/2025mp1795243 Direct URL to data: https://mediatum.ub.tum.de/1795243
Related research article	J. Hofmann, B. Burchard, V. Krieger, K. Wudy, Influence of Geometry-dependent Temperature-Time-Profiles on Density and Dimensional Accuracy in Laser-based Powder Bed Fusion of Polyamide 12, Materials & Design, 259:114,860 (2025), DOI: 10.1016/j.matdes.2025.114860

## 1 Value of the Data

• This dataset provides high-resolution thermal signal data from in-situ IR thermography, directly linked to density and dimensional measurements. It enables systematic analysis of geometry-dependent thermal effects in laser-based powder bed fusion of plastics (PBF LB/P) and includes characteristic thermal features often discussed in the literature for quality prediction and process control.
• Beneficial for additive manufacturing researchers lacking access to industrial thermography setups, and for data scientists or machine learning specialists seeking high-quality, openly accessible datasets. The combined thermal, geometric, and material property data allow algorithm training and benchmarking without requiring specialized hardware.
• Support reuse for predictive model development, benchmarking in-situ monitoring approaches, evaluating geometry-dependent parameter effects, and training adaptive control algorithms. Data are provided as raw signals, processed feature maps, and associated part property measurements in standardized formats to ensure broad applicability.
• Serve as reference data for validating data-driven monitoring strategies, designing new experiments, and extending prior analyses reported in the literature. Combining powder properties, single-layer data, cuboid density, and dimensional accuracy with thermal features enables reproducibility and method comparison across research groups.

## 2 Background

PBF-LB/P enables the fabrication of lightweight, complex components for use across a range of sectors, including the automotive [2], consumer goods [3], and pharmaceutical [4] industries. However, achieving consistent part properties remains a major challenge to its widespread adoption. These challenges arise because the temperature of the melt during laser exposure depends not only on energy input but also on geometry and scan strategy [5]. For instance, in the standard bidirectional scan pattern, laser return times vary with scan vector length, leading to asymmetric temperature–time profiles across the build area [6]. Moreover, larger cross-sections cool more slowly than smaller features, which can promote higher density but also increase the risk of dimensional deviations [7]. Consequently, process parameter selection often requires a trade-off between density and accuracy [8].

Infrared (IR) thermography has emerged as a promising in-situ monitoring approach, capable of capturing spatial and temporal thermal signatures during PBF-LB/P. While emissivity variations and the semi-transparency of polymers such as PA12 complicate absolute temperature determination [9], characteristic features of thermal signals have been correlated with porosity [6], density [10], and mechanical properties [11]. This highlights IR thermography’s potential as a feedback tool for geometry-dependent process control. However, the relationship between temperature–time profiles and dimensional accuracy remains insufficiently explored. Most dimensional accuracy studies in PBF-LB/P have relied on profilometry [12] or fringe projection [13]. Recently, works by Klammert et al. [14] and Greiner [5] showed that IR thermography captures geometry-dependent effects on melt behavior and dimensional accuracy. Interestingly, the peak in the thermal signal and subsequent cooling behavior correlate with porosity [3] and dimensional accuracy [5]. These findings emphasize the potential of IR thermography for closed-loop feedback control systems that compensate for geometry-dependent effects on part properties.

To date, no dataset has consistently linked multiple part properties, such as density and dimensional accuracy, with IR thermography data in PBF-LB/P. The dataset presented here addresses this gap by providing samples with varying energy input, scan speed, hatch distance, part geometry, and their respective thermal histories, density, and dimensional accuracy. Industry practitioners can use this data to design scan strategies that better balance density and dimensional accuracy. However, academics can exploit it to evaluate geometry-dependent parameter effects, benchmark in-situ monitoring approaches, and train adaptive control algorithms.

## 3 Data Description

The dataset in [15] contains raw infrared thermography recordings in a neutral file format, extracted features from the thermal signal, process parameters, build layout information, part properties (density, sample dimensions, and mechanical properties), and figures illustrating thermal feature maps and feature–property relationships.

### 3.1 Folder structure

**Fig. 1:**
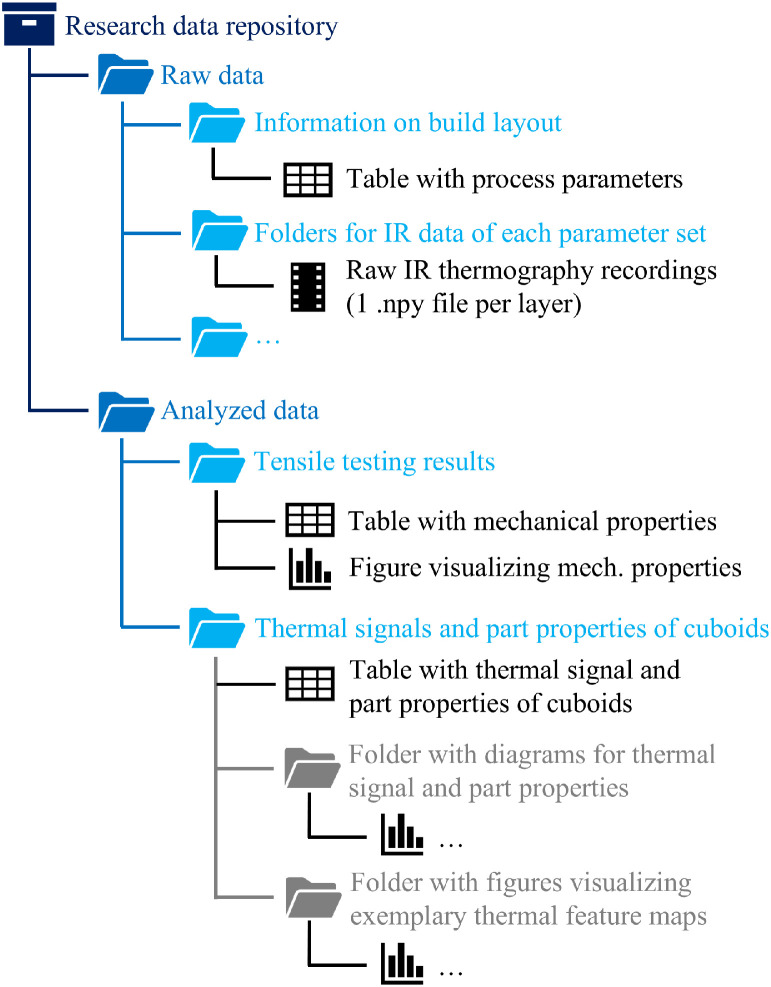
Folder structure of the research data repository in [15].

The folder structure of the research data repository, as visualized in Fig. 1, is organized into two main sections:

1. **Raw data (Size: 0.57** **TB):** This section includes a folder with a table summarizing the process parameters and the build layout. Additionally, it contains folders with the raw infrared thermography recordings stored as .npy files, one for each layer, structured into subfolders according to the respective process parameter sets.
2. **Analyzed data (Size: 57** **MB):** This section consists of two parts. The first folder contains tensile testing results, including a table with the measured mechanical properties and corresponding visualizations. The second folder provides the cuboid samples' thermal signals and part properties, which are listed in a table and organized in subfolders. These subfolders include diagrams illustrating the relationships between thermal signals and part properties, and figures showing exemplary thermal feature maps of the last layer of each sample.

### 3.2 Raw data

#### 3.2.1 Information to build layout

The Excel file “Information to Build Layout.xlsx” provides additional information on the build layout. It contains the following information:

• Process parameters used for cuboid and tensile test samples.
• Components in the field of view of the IR camera, listed in order of exposure.
• Screenshots of the build layout created in the slicer software (Autodesk, Netfabb).
• Samples in the field of view of the IR camera, including their size, process parameters, and the absolute layer number within the build process, in which the last layer of each sample was exposed.
• Components manufactured in parallel but outside the IR camera field of view.

#### 3.2.2 Uncalibrated infrared thermography data

**Fig. 2:**
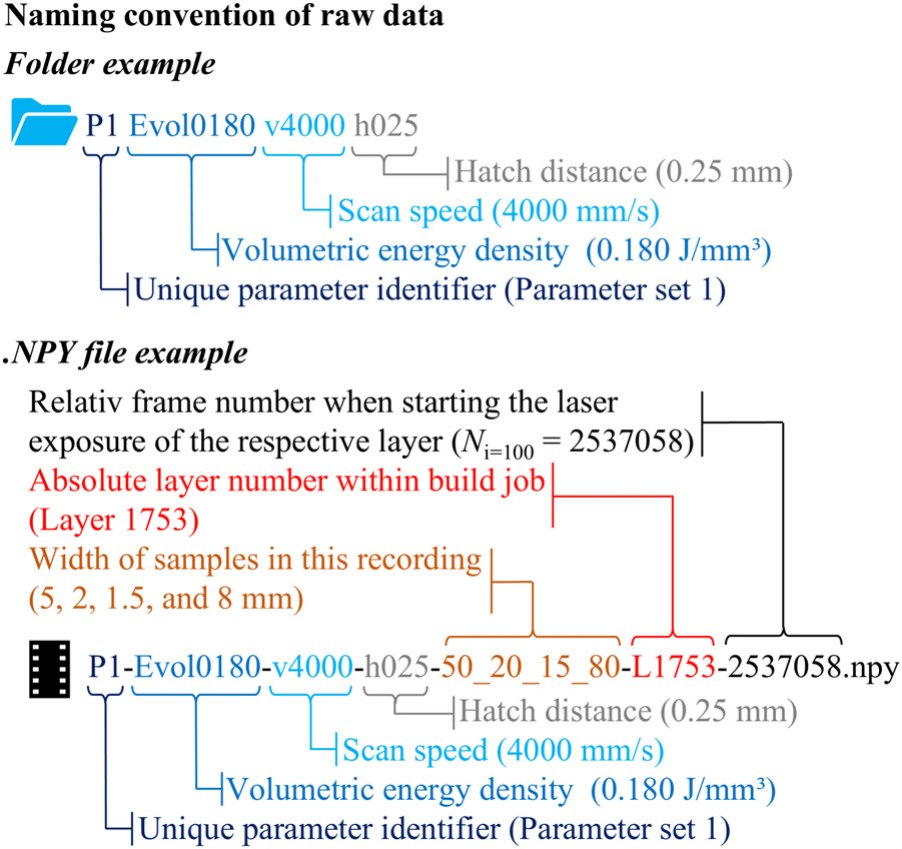
Naming convention of folders and .npy-files containing the raw IR thermography data.

IR thermography recordings are organized in folders according to the process parameters used. The naming convention of the raw data folders encodes the parameter set with a unique identifier (P1–P8) along with the corresponding volumetric energy density, scan speed, and hatch distance, as shown in Fig. 2. The recordings themselves follow the same naming convention but additionally include the width of the samples shown in the recording. Each file contains the thermal data of one layer, and the naming convention specifies the absolute layer number according to the build layout. For each sample, the top six layers are provided.

The uncalibrated thermal data are supplied as standardized NumPy (.npy) files, a common format in Python that facilitates reuse. All files have the format (N_Frame_,X_px_,Y_px_), where X_px_ and Y_py_ represent the pixel coordinates of the thermal values, and N_Frame_ is the frame index in the recording.

In each recording, laser exposure begins at N_Frame_ = 100. Frames before this point capture the powder bed temperature before laser irradiation. The recording duration was chosen so the acquisition ends shortly before or after the recoating step. Thermal data covering the time from recoating to the start of laser exposure of the subsequent layer are not included.

For each recording, the respective layer time t_layer_ can be calculated from the camera frame rate (200 Hz) and the index number of frame N_i=100_, which are also part of the file naming convention:

(1) $$t_{\text{layer}\;n}=\left(N_{i=100,n+1}-N_{i=100,n}\right)\;\frac{1}{200}$$

**Fig. 3:**
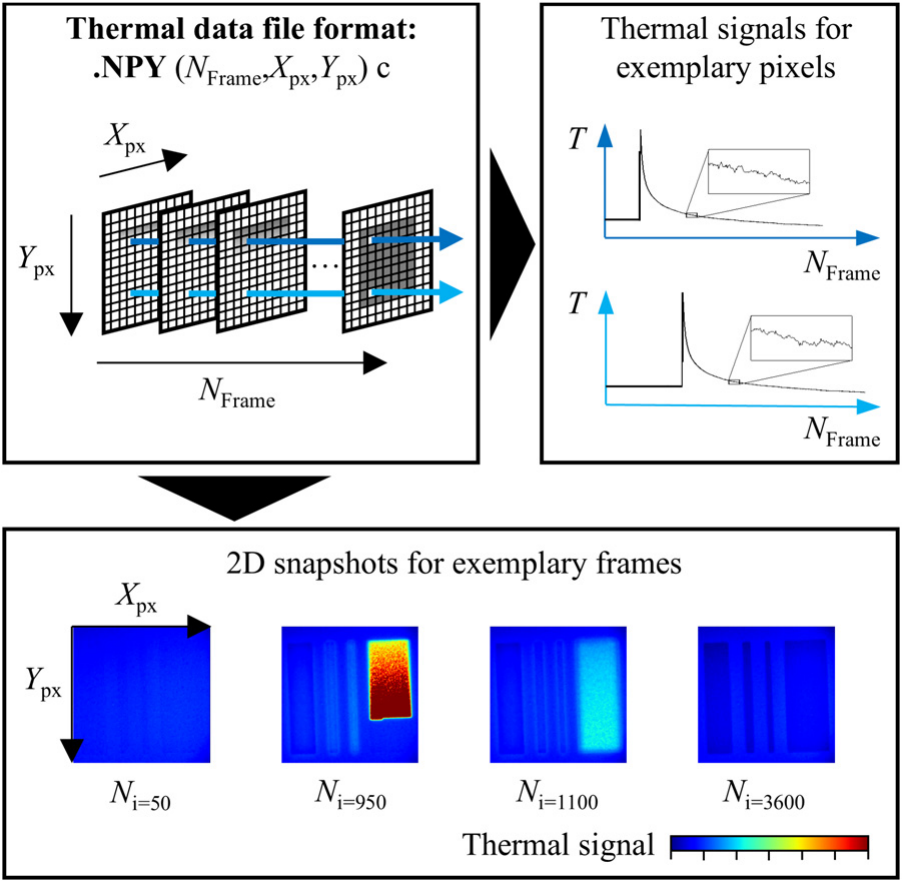
Raw infrared thermography file format and exemplary visualizations of temperature-time profiles of pixels and 2D snapshot images of frames.

Here, the layer time is defined as the interval between the start of laser exposure N_i=100_ of layer n and that of the subsequent layer *n* + 1. The frame indices are relative values based on the camera's internal clock. Since this clock may have been reset during recording, t_layer_ can only be calculated within a sample, but not across different samples. Fig. 3 illustrates the file format and 2D thermal frames from one recording.

### 3.3 Analyzed data: thermal signals and part properties of cuboid samples

The table “Thermal Signals and Part Properties Cuboids.xlsx” contains thermal features (derived from IR recordings) and part properties of the cuboid samples and single layers. For each sample and process parameter combination, mean values are provided along with their standard deviation across the top six layers. The dataset includes:

• Fit variable a [ °C·s]
• Fit variable b [s]
• Fit variable c [–]
• Fit variable d [ °C]
• Thermal signal pre-exposure [ °C]
• Peak of thermal signal [ °C]
• Peak of thermal signal filtered for pixels in laser spot [ °C]
• Thermal signal at decay [ °C]
• Decay time at –1 K/s [s]
• Thermal signal rise of the powder coated on the melt [K]
• Sample width [mm]
• Sample length [mm]
• Sample height [mm]
• Sample mass [g]
• Volumetric density (mass/sample dimensions) [g/cm³]
• Archimedean density [g/cm³]
• Single layer thickness (melt depth) [mm]

This list provides an overview of commonly reported values in the literature. For instance, the fit variables enable reconstruction of the average temperature–time profile described by Hofmann et al. [16]. Other researchers can develop improved data compression techniques for reconstructing temperature–time profiles, or extract additional features as proposed in [6].

#### 3.3.1 Spatial temperature distribution of the melt during processing

**Fig. 4:**
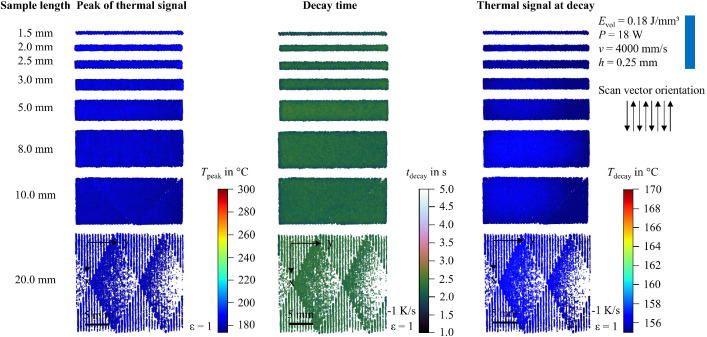
Influence of the sample width on the peak of the thermal signal, decay time, and thermal signal at decay at an energy density of 0.18 J/mm³ (4000 mm/s).

**Fig. 5:**
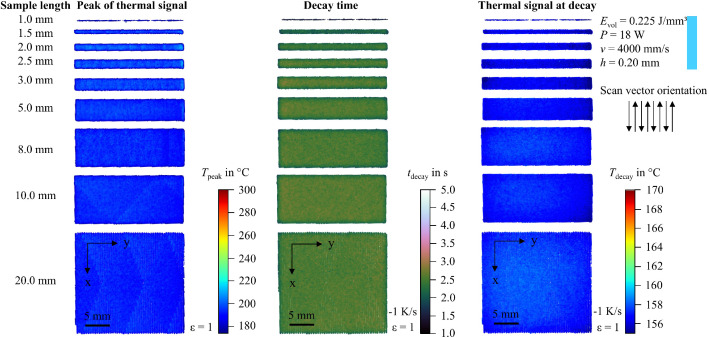
Influence of the sample width on the peak of the thermal signal, decay time, and thermal signal at decay at an energy density of 0.225 J/mm³ (4000 mm/s).

**Fig. 6:**
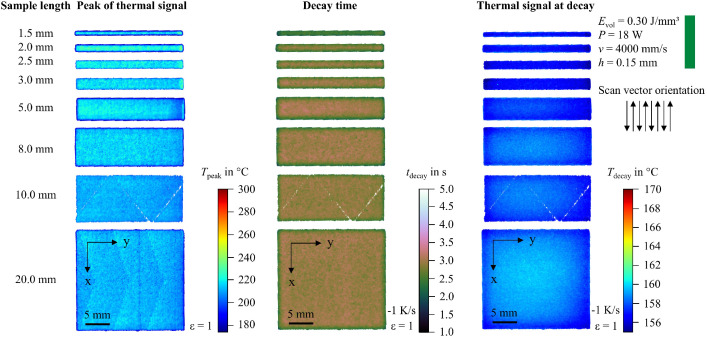
Influence of the sample width on the peak of the thermal signal, decay time, and thermal signal at decay at an energy density of 0.30 J/mm³ (4000 mm/s).

**Fig. 7:**
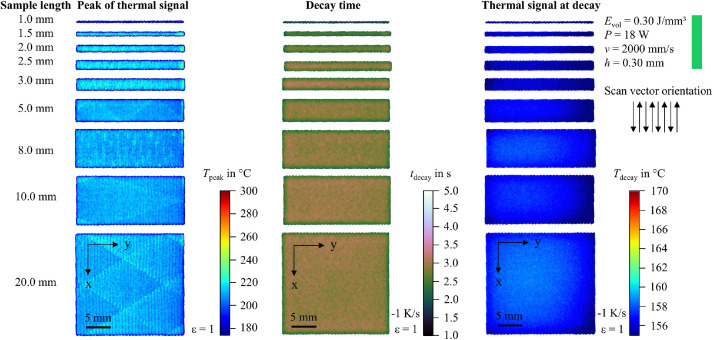
Influence of the sample width on the peak of the thermal signal, decay time, and thermal signal at decay at an energy density of 0.30 J/mm³ (2000 mm/s).

**Fig. 8:**
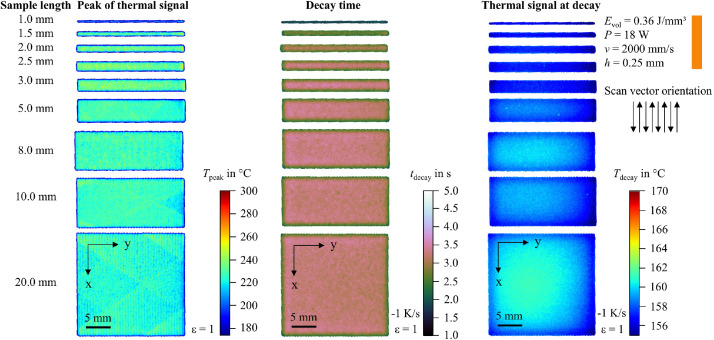
Influence of the sample width on the peak of the thermal signal, decay time, and thermal signal at decay at an energy density of 0.36 J/mm³ (2000 mm/s).

**Fig. 9:**
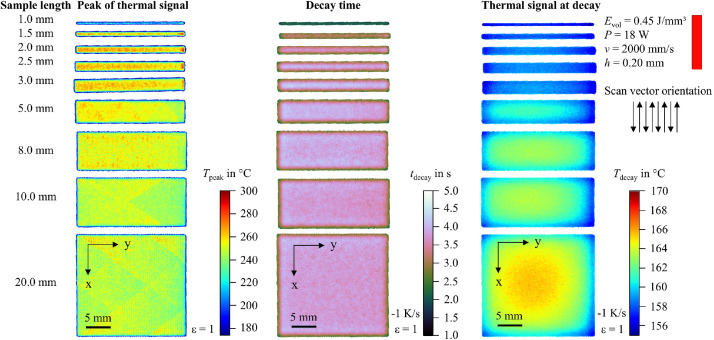
Influence of the sample width on the peak of the thermal signal, decay time, and thermal signal at decay at an energy density of 0.45 J/mm³ (2000 mm/s).

**Fig. 10:**
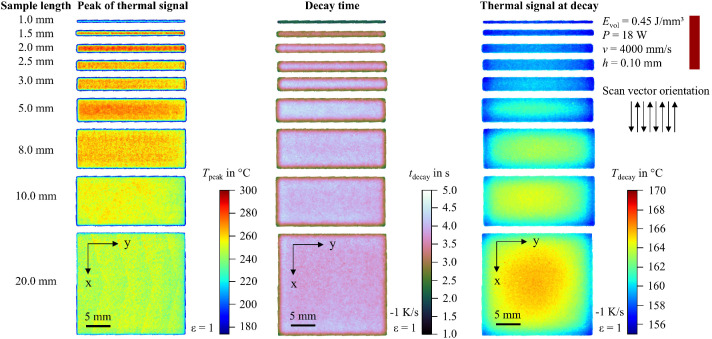
Influence of the sample width on the peak of the thermal signal, decay time, and thermal signal at decay at an energy density of 0.45 J/mm³ (4000 mm/s).

**Fig. 11:**
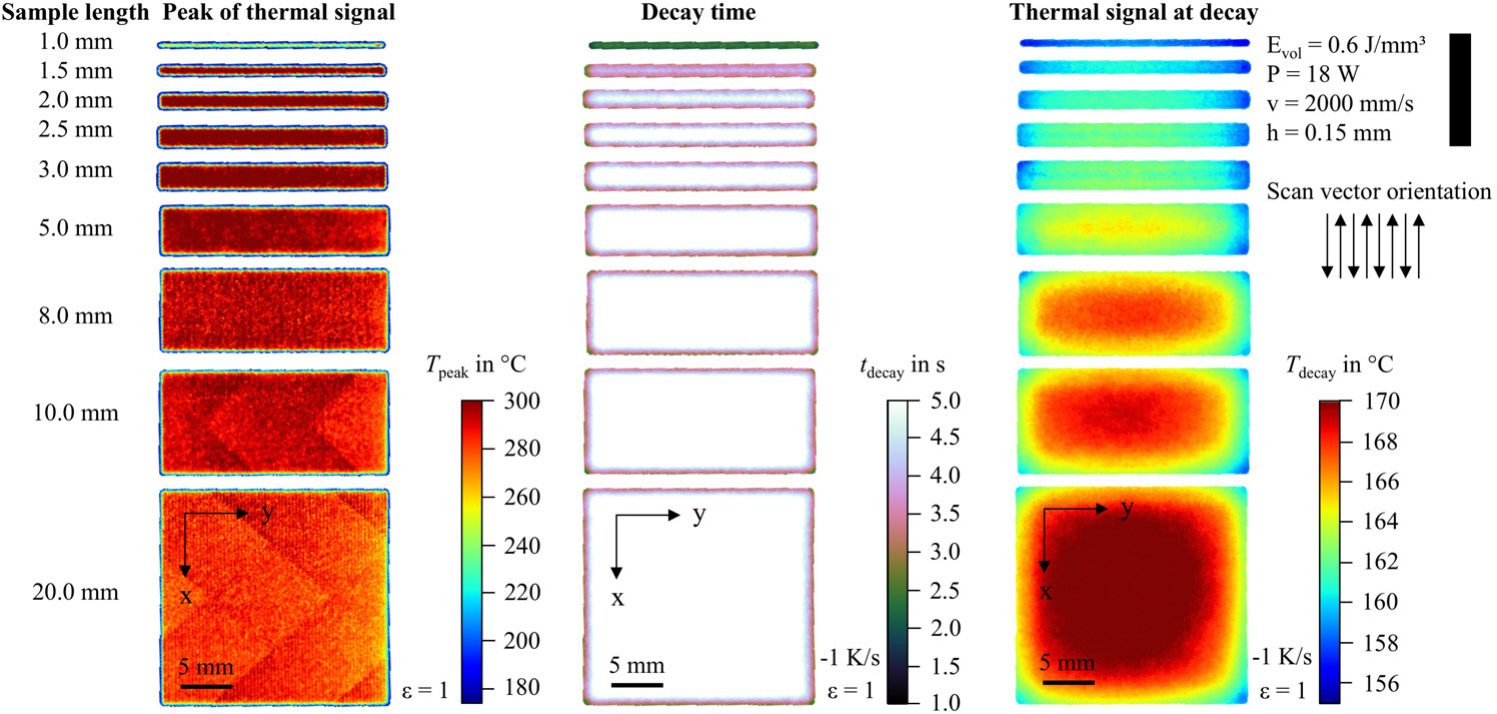
Influence of the sample width on the peak of the thermal signal, decay time, and thermal signal at decay at an energy density of 0.60 J/mm³ (2000 mm/s).

In addition to the snapshot images shown in Fig. 3, the raw data can be visualized in multiple ways. For example, the peak value of the thermal signal at each pixel can be integrated into a single image. To support researchers in navigating the dataset and identifying recordings of interest, 2D feature maps of exemplary thermal signals in the last layer are included and shown in Fig. 4, Fig. 5, Fig. 6, Fig. 7, Fig. 8, Fig. 9, Fig. 10, Fig. 11. These figures include the peak of the thermal signal, decay time, and thermal signal at decay for all sample sizes and process parameters. These features are commonly discussed in the literature [5,6,11], demonstrating the broad usability of the dataset. The naming convention in the repository follows the folder structure described before.

#### 3.3.2 Diagrams for thermal signal and part properties

**Fig. 12:**
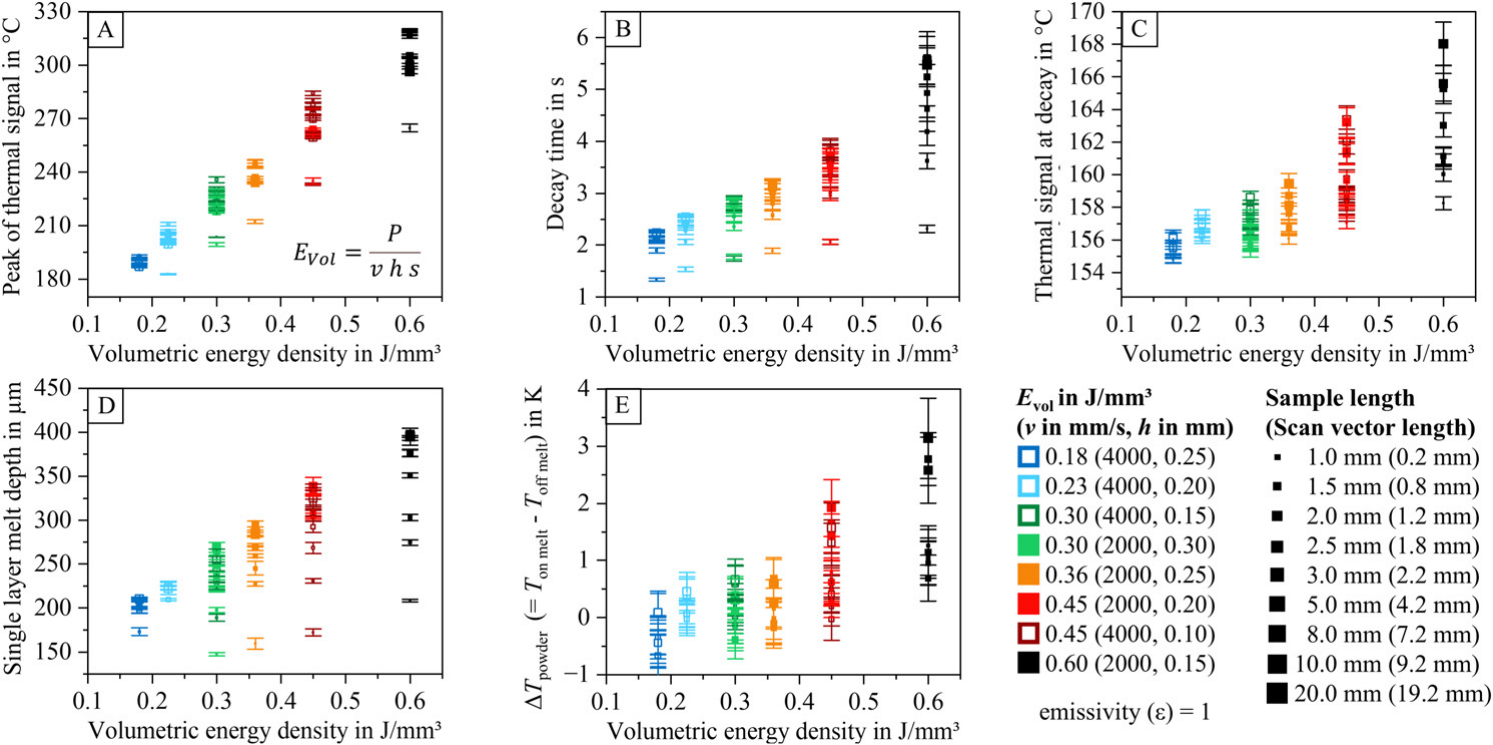
Influence of volumetric energy density on the peak of the thermal signal (A), decay time (B), thermal signal at decay (C), single layer melt depth (D), and thermal signal rise of the powder coated on the melt (E) for samples manufactured with varying scan vector length and scan parameters.

**Fig. 13:**
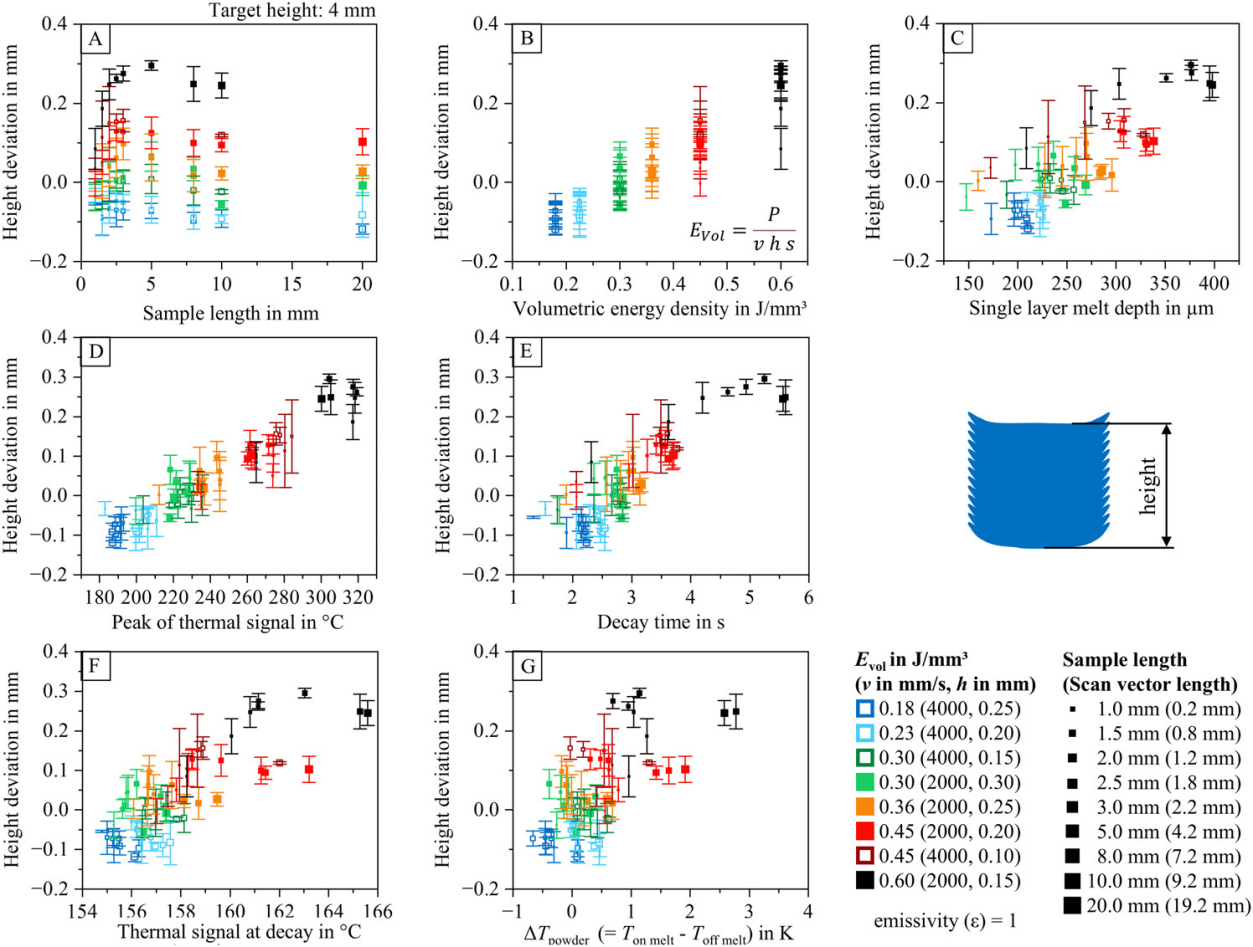
Deviation from the target height of 4 mm as a function of sample width (A), volumetric energy density (B), single layer melt depth (C), peak of the thermal signal (D), decay time (E), thermal signal at decay (F), and thermal signal rise of the powder coated on the melt (G) for samples manufactured with varying scan vector length and scan parameters (utilizing a z-compensation of 0.1 mm).

**Fig. 14:**
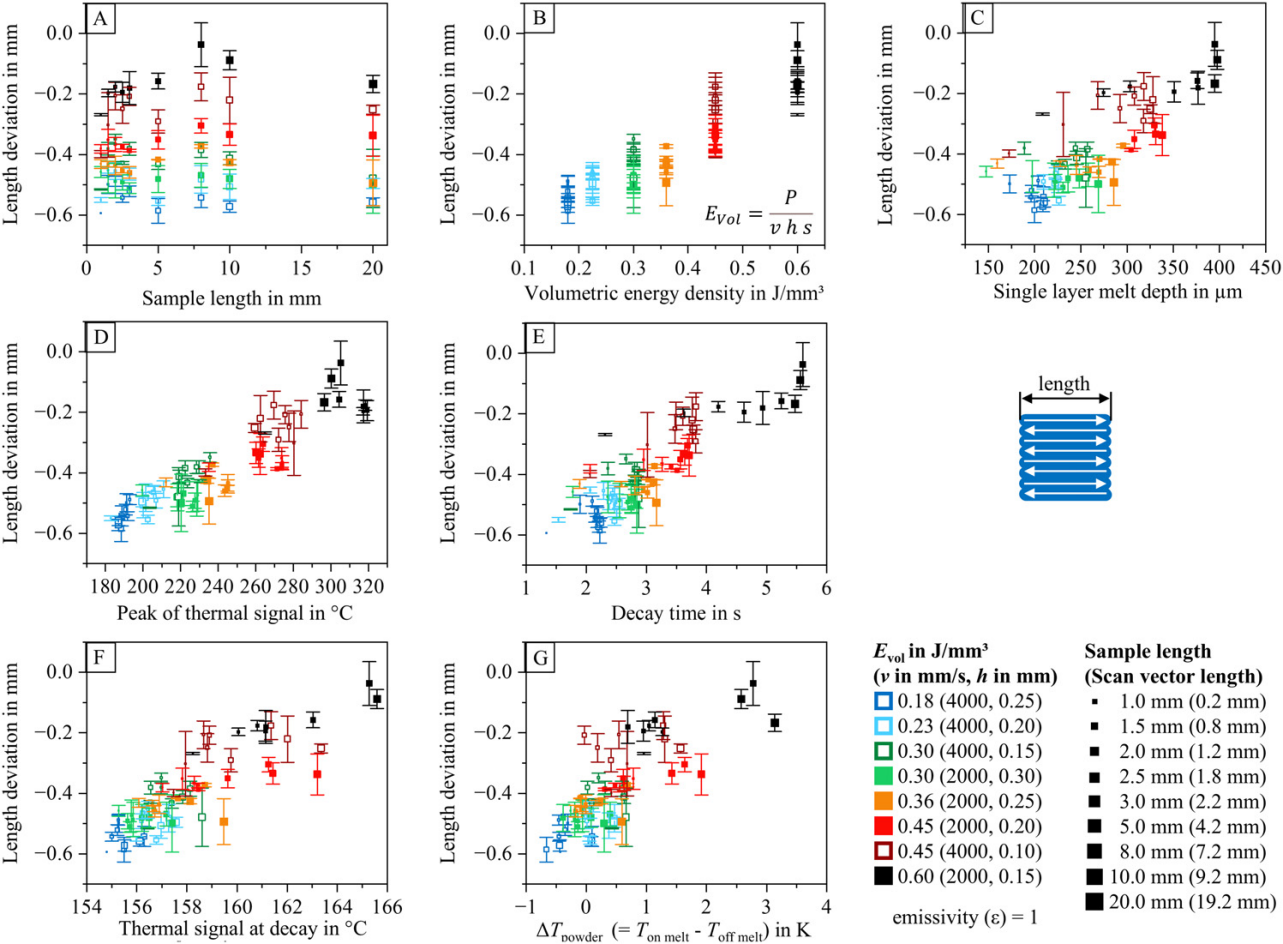
Deviation from the target sample length (along the scan vector) as a function of sample width (A), volumetric energy density (B), single layer melt depth (C), peak of the thermal signal (D), decay time (E), thermal signal at decay (F), and thermal signal rise of the powder coated on the melt (G) for samples manufactured with varying scan vector length and scan parameters (utilizing a total offset of 0.40 mm between the part’s contour and the scan vectors (contour offset: 0.25 mm; hatch offset: 0.15 mm).

**Fig. 15:**
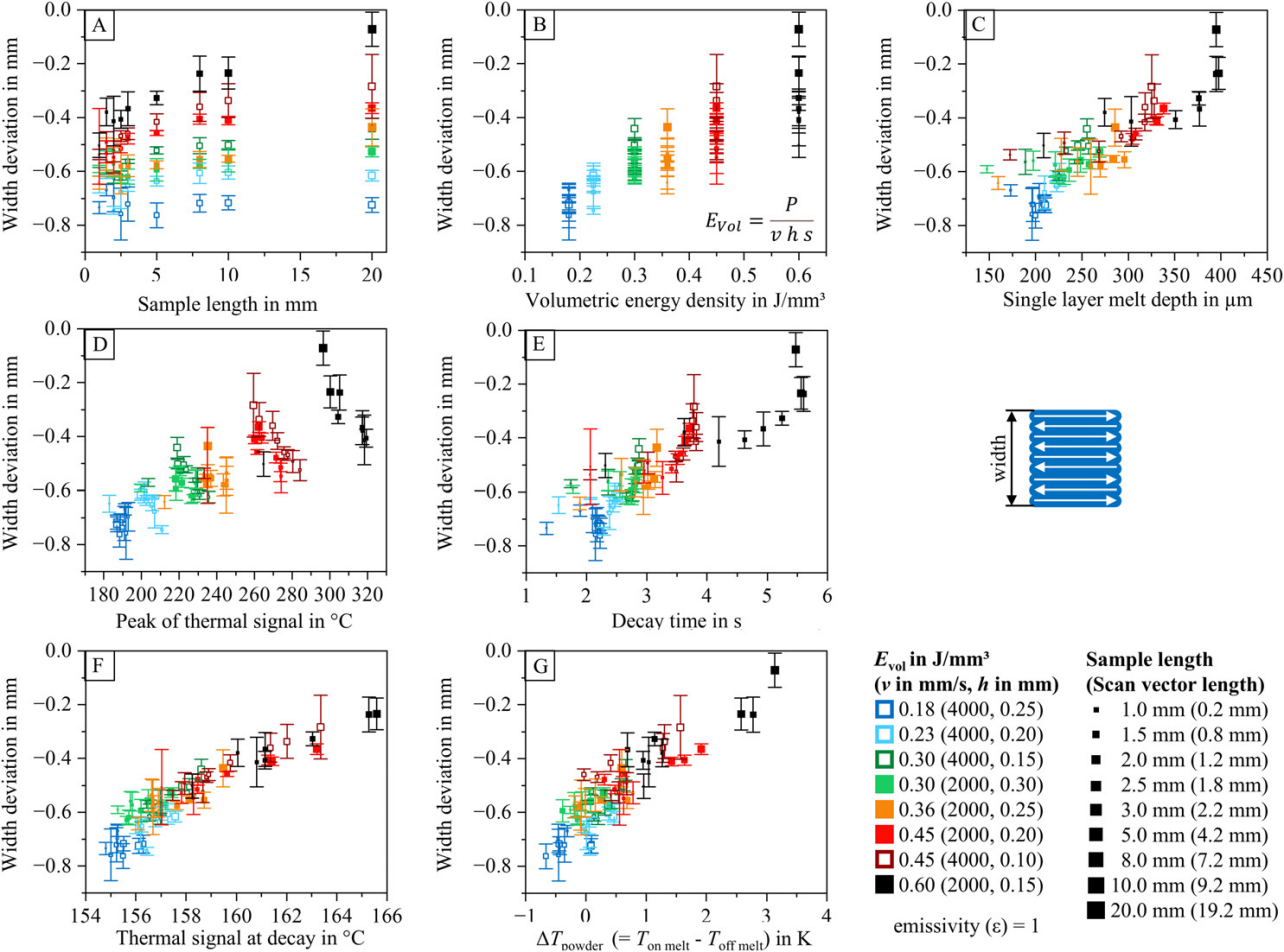
Deviation from the target width of 20 mm (perpendicular to scan vectors) as a function of sample width (A), volumetric energy density (B), single layer melt depth (C), peak of the thermal signal (D), decay time (E), thermal signal at decay (F), and thermal signal rise of the powder coated on the melt (G) for samples manufactured with varying scan vector length and scan parameters (utilizing a total offset of 0.40 mm between the part’s contour and the scan vectors (contour offset: 0.25 mm; hatch offset: 0.15 mm).

**Fig. 16:**
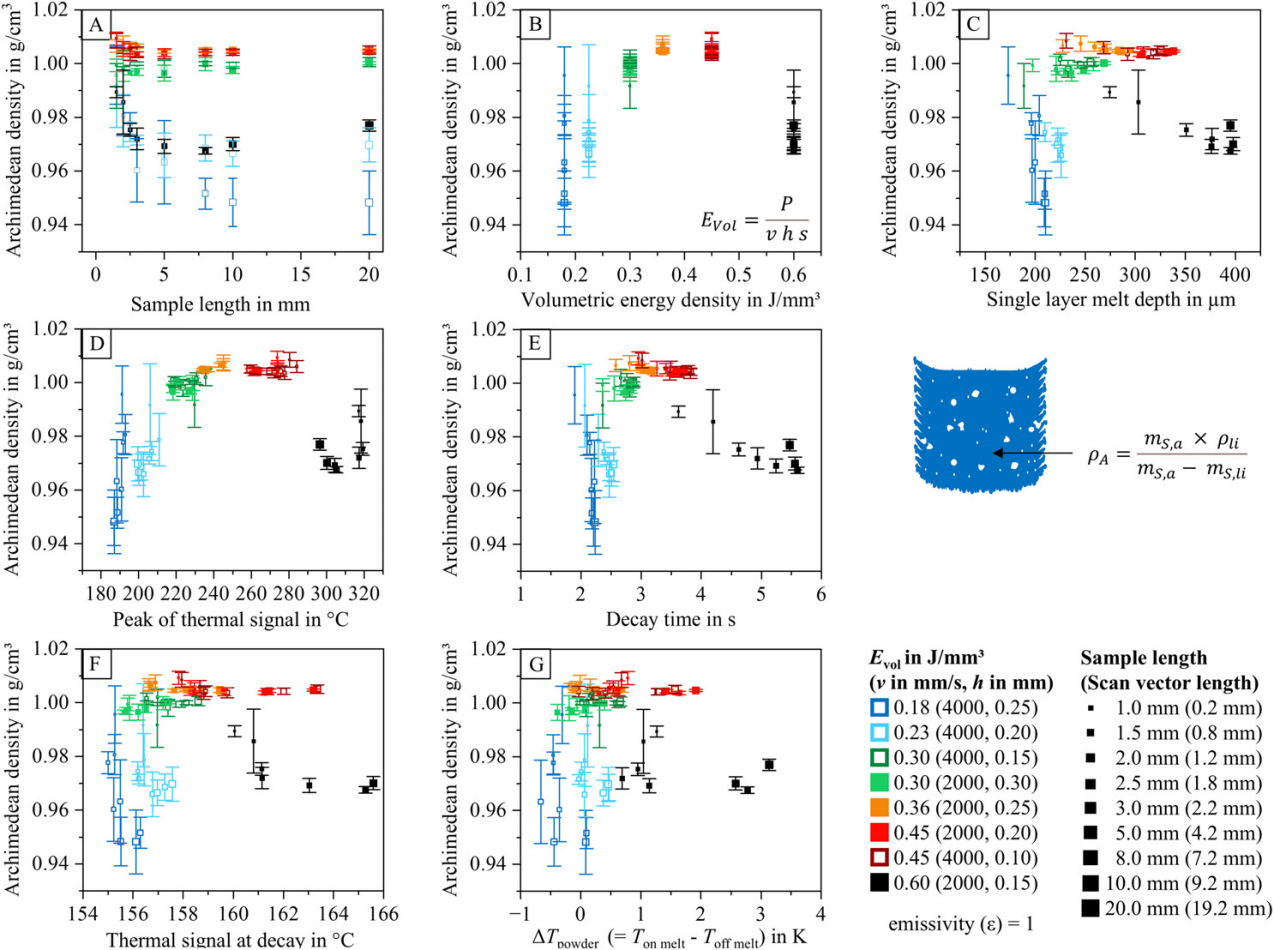
Archimedean density as a function of sample width (A), volumetric energy density (B), single layer melt depth (C), peak of the thermal signal (D), decay time (E), thermal signal at decay (F), and thermal signal rise of the powder coated on the melt (G) for samples manufactured with varying scan vector length and scan parameters.

**Fig. 17:**
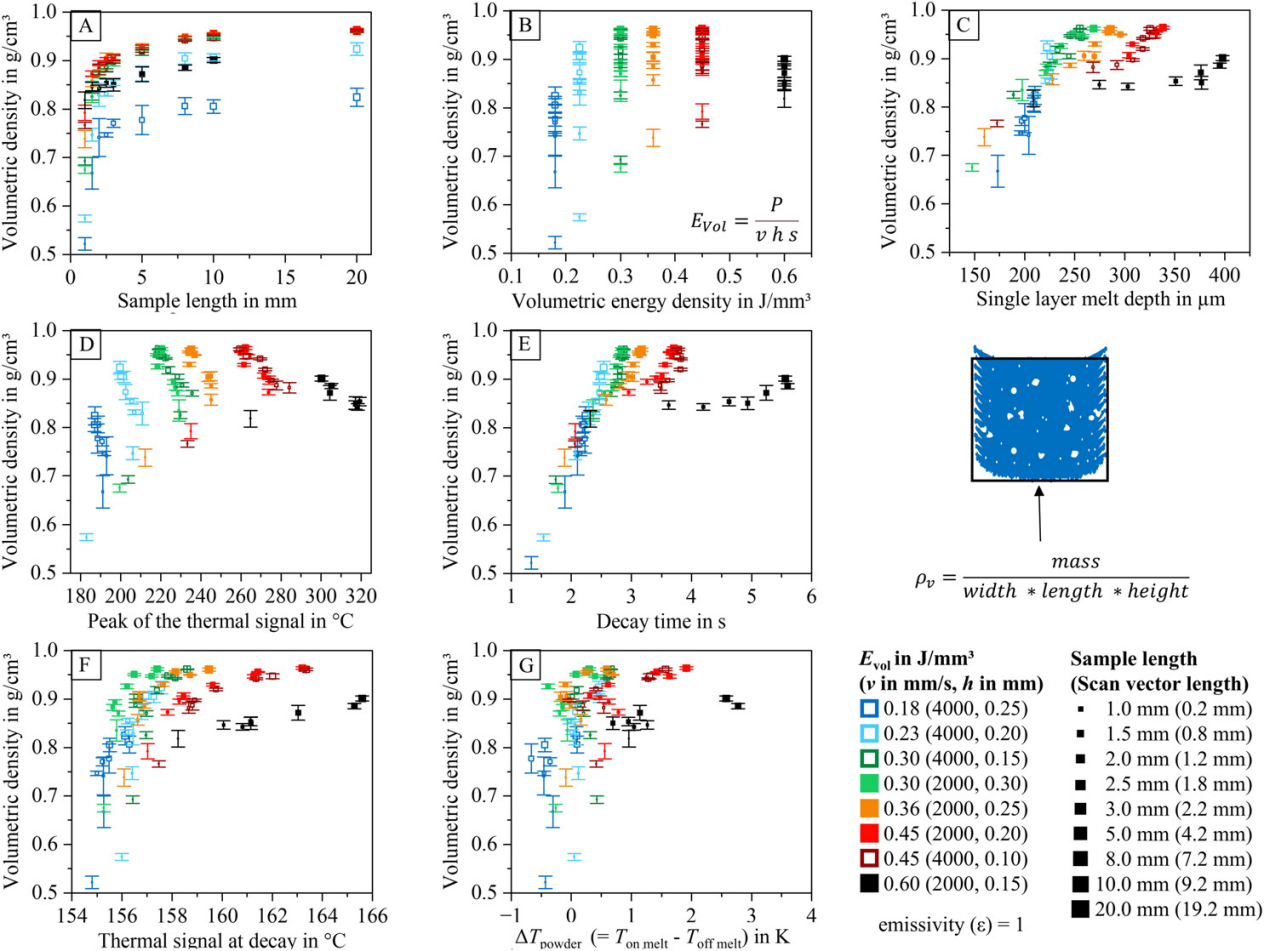
Volumetric density (calculated from sample mass and its width, length, and height) as a function of sample width (A), volumetric energy density (B), single layer melt depth (C), peak of the thermal signal (D), decay time (E), thermal signal at decay (F), and thermal signal rise of the powder coated on the melt (G) for samples manufactured with varying scan vector length and scan parameters.

To help researchers navigate the dataset and identify relevant information within the analyzed data table (containing 2886 entries), diagrams visualizing relationships between process parameters, part properties, and thermal features are provided. These diagrams are included in the dataset and illustrated in Fig. 12, Fig. 13, Fig. 14, Fig. 15, Fig. 16, Fig. 17. The naming conventions correspond to the content of the figures, covering:

• Influences of volumetric energy density
• Influences on sample height
• Influences on sample width
• Influences on sample length
• Influences on Archimedean density
• Influences on volumetric density (mass/sample dimensions)

The visualized relationships include sample width, volumetric energy density, single-layer melt depth, peak thermal signal, decay time, thermal signal at decay, and the thermal signal rise of the powder layer on the melt. Data points are shown for each process parameter set and sample size combination.

### 3.4 Analyzed data: mechanical properties

**Fig. 18:**
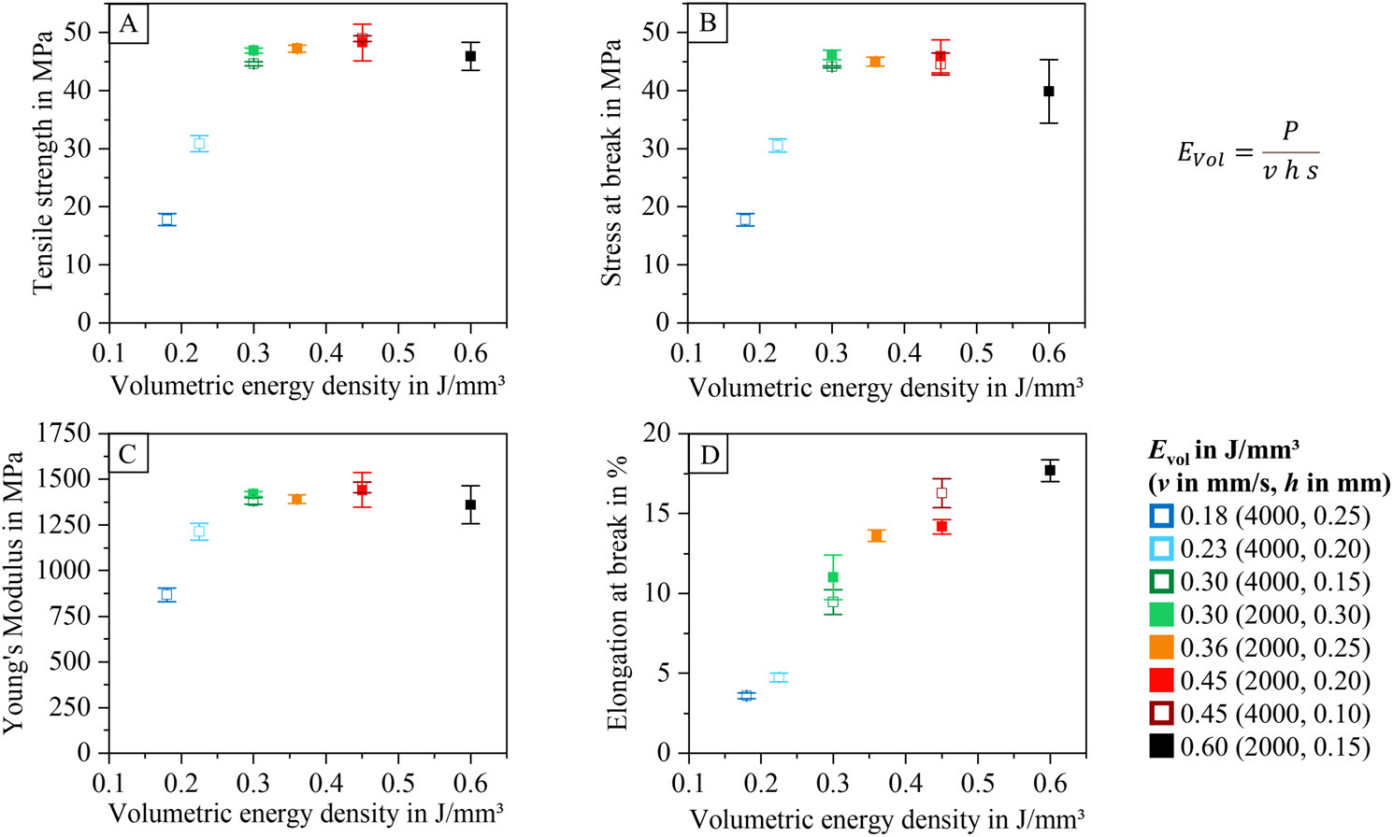
Tensile strength (A), stress at break (B), Young's modulus (C), and elongation at break (D) for samples produced with varying laser and scan parameters.

Tensile test samples were produced alongside the cuboid samples, but were not monitored with the infrared thermography camera. The tensile strength, elongation at break, stress at break, and Young’s modulus are summarized in the table “Mechanical Properties.xlsx”. Diagrams visualizing these results are included in the dataset and shown in Fig. 18.

## 4 Experimental Design, Materials and Methods

The methodology presented in this chapter follows the approach detailed in the associated co-publication [1]. The following sections present a systematic reproduction of that work, restated with additional context and precision for clarity, while maintaining the original study's technical structure and data organization. Further information, such as the absolute layer numbers and exact sample positions within the build job, is available in the “Information to Build Layout.xlsx” included in the raw data folder of the associated repository [15].

Please note that this dataset does not discuss effects at sample edges related to system-specific hardware or software, such as those arising from the Gaussian intensity distribution of the CO₂ laser, laser rise and fall times, calibration errors, or sky-writing strategies. These edge effects can influence the local energy input but are highly system-dependent. For the PBF-LB/P system used here, these settings are pre-calibrated, non-adjustable, and proprietary information of the system manufacturer. A detailed discussion of the laser dynamics and calibration influences is available in our co-publication [1]. Furthermore, it should be noted that due to the infrared camera’s limited frame rate (200 Hz) and spatial resolution (75 µm/pixel), these localized edge effects related to system-specific hardware or software may not be captured in the thermal data.

### 4.1 Powder preparation and machine

The experiments were conducted on an industrial PBF-LB/P system (Formiga P100, EOS) using a commercially available polyamide 12 (PA12) powder (PA2200, EOS). To replicate common industrial practice, virgin powder was mixed with used powder in a 1:1 mass ratio utilizing the manufacturer’s unpacking and sieving station.

Before processing, the powder was characterized to verify its suitability for robust layer deposition and laser exposure. Bulk and tapped densities were measured, and the Hausner ratio was calculated to verify free-flowing behavior. Rheological properties of the melt were further assessed via melt volume rate (MVR) measurements with a melt flow indexer (Mflow, Zwick Roell) following DIN EN ISO 1133 at 230 °C under a 2.16 kg load. The results are summarized in Table 1.

**Table 1:** Powder characterization results.

	1:1 blend (used for experiment)	Virgin (for reference)	Method / Standard
Bulk density in g/cm³	0.428 ± 0.007	–	DIN EN ISO 60
Tapped density in g/cm³	0.518 ± 0.009	–	Manual tapping of 200 ml cylinder until a volume change < 2 ml/min
Hausner ratio	1.21 ± 0.03	–	(= tapped density / bulk density)
Melt volume rate in cm³/10 min	39.5 ± 4.7	53.2 ± 5.0	DIN EN ISO 1133 (230 °C, 2.16 kg)

### 4.2 Experimental setup

Rectangular cuboids were fabricated (length: 1 – 20 mm, height: 4 mm, width: 20 mm) to examine the influence of geometry-dependent temperature–time profiles on density and dimensional accuracy. Furthermore, single-layer samples were fabricated to investigate how variations in overlap ratios and maximum laser return times influence melt depth during hatching. Note that no thermal data is provided for the single layers in this dataset.

**Fig. 19:**
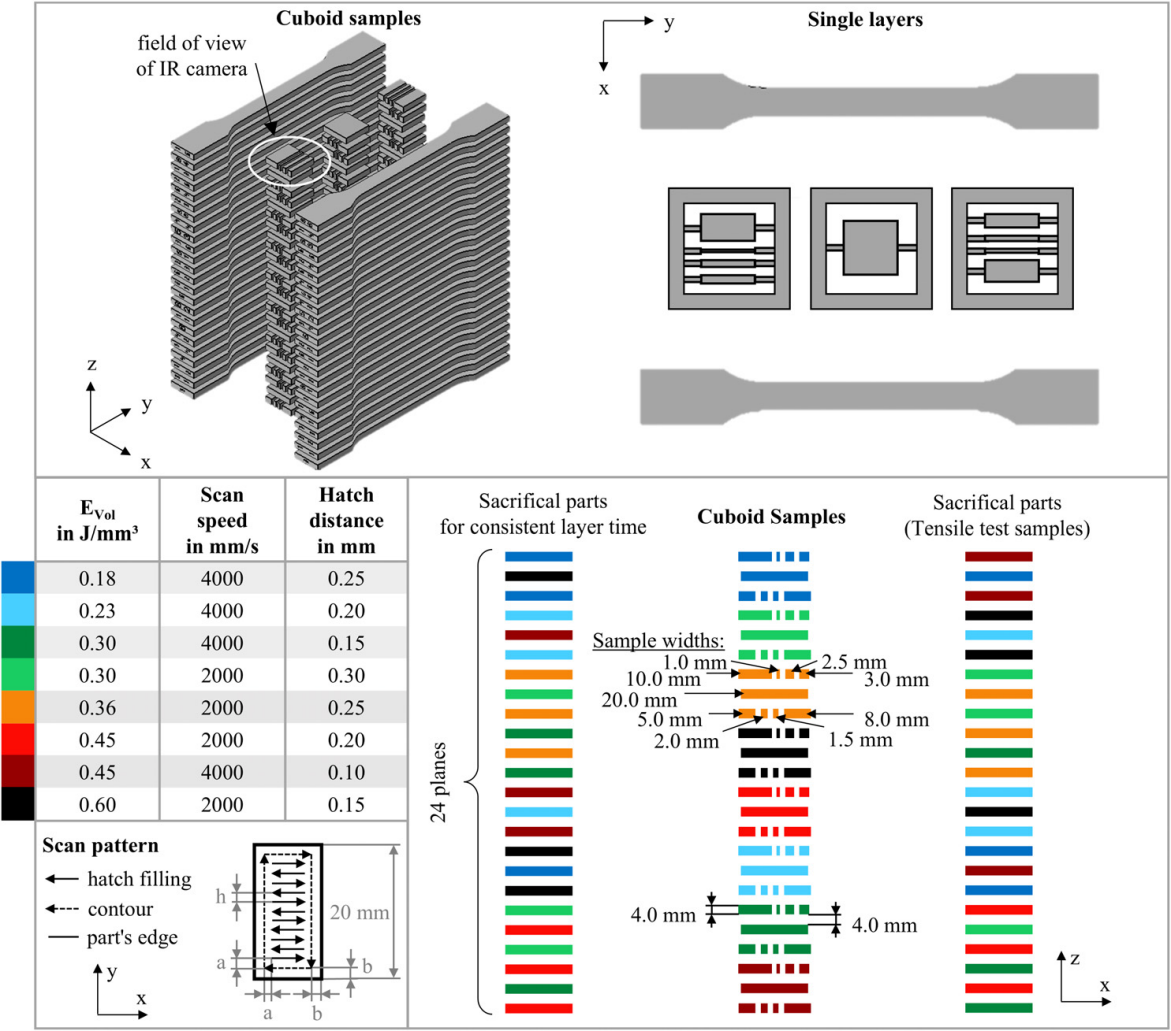
Visualization of the build layout of the cuboid samples, single layers, tensile test samples, and the industry-standard scan pattern, including the processing parameters used. Adopted from [1].

Hatch distances were varied between 0.10 and 0.30 mm. The resulting volumetric energy densities were maintained between 0.18 and 0.60 J/mm³ to achieve complete fusion without inducing smoke formation. Additionally, the process parameters summarized in Table 2 were selected to allow evaluation of energy density and the interaction between scan speed and hatch distance at fixed energy densities of 0.30 and 0.45 J/mm³. A bidirectional scan pattern was used for laser exposure, as illustrated in Fig. 19. For each combination of scan vector length and process parameters, three samples were produced (3 samples × 8 parameter sets × 8 scan vector lengths = 192 total samples). The build layout is shown in Fig. 19.

**Table 2:** Laser and scan parameters used.

E_Vol_ in J/mm³	Scan speed in mm/s	Hatch distance in mm	Laser power in W	Layer height in mm
0.18	4000	0.25	18	0.1
0.23	4000	0.20	18	0.1
0.30	4000	0.15	18	0.1
0.30	2000	0.30	18	0.1
0.36	2000	0.25	18	0.1
0.45	2000	0.20	18	0.1
0.45	4000	0.10	18	0.1
0.60	2000	0.15	18	0.1

The scan vectors were aligned to the sample length, allowing separate analyses of dimensional deviations parallel and perpendicular to the scan direction. Contour exposure was omitted, but a consistent 0.8 mm offset between the hatch-filling area and the part edge was applied, ensuring that the scan vector length scaled proportionally with the ratio of hatch-filling to total sample area, consistent with industry practice.

Because samples were heated near the polymer’s melting temperature range during processing, scaling factors were applied to compensate for shrinkage due to crystallization and thermal contraction during cooling to ambient temperature. The scaling factors, determined according to the machine manufacturer’s calibration guidelines, are provided in Table 3 along with all other relevant process parameters and sample dimensions. As a result of scaling, the actual scan vector lengths for cuboids and single layers were slightly larger during processing (see Table 4). However, all analyses and reported dimensions use the theoretical (pre-scaled) lengths for simplicity.

**Table 3:** Additional process parameters and sample geometries used.

Processing temperatures	170/148 °C (surface/chamber)
**Sky-writing**	on
**Contour power**	0 W (off)
**Contour offset (b)**	0.25 mm
**Hatch offset (a)**	0.15 mm
**Scaling** **(thermal expansion)**	X: 3.1 % Y: 3.0 % Z(0 mm): 2.2 % Z(300 mm): 1.6 %
**z-compensation**	0.1 mm
**Sample width**	20 mm
**Sample height**	4 mm

**Table 4:** Sample length and corresponding scan vector length.

**Sample length in mm**	1.0	1.5	2.0	2.5	3.0	5.0	8.0	10.0	20.0
**Scan vector length in mm**	0.2	0.7	1.2	1.7	2.2	4.2	7.2	9.2	19.2

A minimal layer time cannot be explicitly set on the used PBF-LB/P system. Instead, the layer duration is determined by the time required to scan each layer. To homogenize layer time across varying scan parameters, tensile test samples were produced as sacrificial parts alongside the cuboids but outside the field of view of the IR camera. All parts were evenly distributed within the build volume to reduce positional effects on part properties. This layout, which is visualized in Fig. 19, resulted in a uniform layer time of 42.3 ± 1.8 s, comparable to industrial standards. A labeled box was manufactured around the cuboid samples of each parameter set and sample position to facilitate the removal of samples from the build volume. This was necessary because the cuboid samples themselves lacked labeling. Comprehensive details are available in the “Information to Build Layout.xlsx” file, which is included in the raw data folder of the associated repository [15]. After fabrication, the cuboid specimens, single layers, and tensile test samples were manually cleaned by sandblasting (Dentablast Duo, Dentaurum) with glass beads at 6 bar pressure.

### 4.3 Infrared thermography process monitoring for raw data generation

**Fig. 20:**
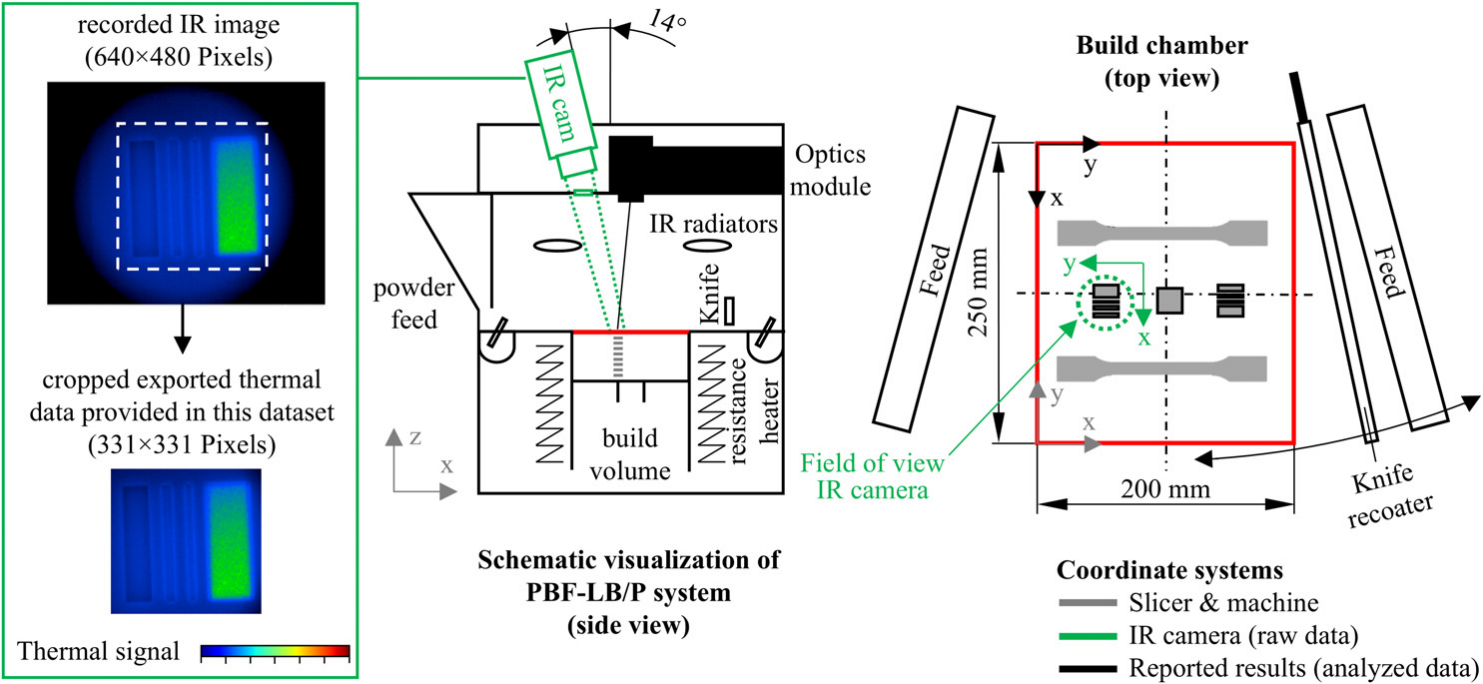
Schematic visualization of the front view of the machine and the top view of the build chamber of the PBF-LB/P system used, as well as the respective coordinate systems.

Process monitoring was carried out using a mid-wave infrared camera (ImageIR 8300, InfraTec GmbH). The system operates in the spectral range of 1.5 – 5.7 µm with a detector resolution of 640×480 pixels and an acquisition rate of 200 Hz. To prevent reflections of the CO_2_ laser, the optical path was shielded with a sapphire window (#48–922, Edmund Optics). The camera was aligned with a tilt of approximately 14° relative to the vertical axis of the machine. For clarity, the inclination is depicted along the y-axis of the IR camera coordinate system in the schematic visualization of the PBF LB/P setup shown in Fig. 20. In the actual configuration, however, the camera was mounted at an inclination in the x-direction, directly in front of the scanner unit. The focal plane was calibrated at the center of the observed region. Therefore, areas farther from the center of the raw data frames may appear slightly defocused in the x direction of the IR camera coordinate system. The effective resolution on the powder bed corresponds to about 75 µm/pixel. The observable field of view is limited to a circular region with a diameter of about 35 mm positioned near the center of the build plane (200×250 mm), as the sapphire window needed to be mounted close to the camera lens. Thus, the raw images in the dataset were cropped to 331×331 pixels to exclude regions obscured by the window mount, significantly reducing file size.

Data acquisition was automated by a software trigger, which initiated recording once the powder bed temperature crossed a threshold value adjusted to coincide with the onset of laser exposure. The internal buffer ensured that 99 frames before this event were preserved, enabling evaluation of the powder bed immediately before exposure. In each dataset, laser scanning begins at frame index *N* = 100. The recording length was set such that the recording ended just before or shortly after recoating. No thermal information was collected between recoating and the next exposure.

As noted in the limitations section, PA12 exhibits partial transparency in the MWIR range. Consequently, subsurface contributions can distort the thermal signal in the earliest layers. Only the upper six layers are included in the dataset to mitigate this effect. In addition, the recorded signal depends on several factors specific to the monitoring setup, such as the preset powder bed temperature, viewing angle, and emissivity. Reported values are therefore provided as relative indicators of thermal behavior rather than calibrated absolute temperatures. All data are given in °C, assuming an emissivity of 1.

Each thermal frame was exported as a 2D matrix representing pixel values for reuse and analysis. Subsequently, the whole sequence of frames was compiled into NumPy arrays using Python. The resulting files follow the structure (N_Frame_, X_px_, Y_px_), where N_Frame_ corresponds to the temporal index with a 5 ms step size. X_px_ and Y_px_ represent the spatial pixel coordinates. It should be noted that the coordinate systems of the slicer, machine, and camera differ, as illustrated in Fig. 20.

### 4.4 Thermal data processing for analysis

The first step of the analysis involves extracting the peak of the thermal signal (T_peak_) from each pixel’s temperature–time profile. This metric corresponds to the highest temperature reached during laser exposure. A limitation is that the moving laser beam covers roughly 20 mm per frame at a scan speed of 4000 mm/s, which introduces a minor distortion in images that visualize T_peak_ distribution. Therefore, only pixels coinciding with the laser position at the moment of frame acquisition are considered when calculating the mean of T_peak_ within a sample. The ratio R_pixel__%_ between the laser spot area A_spot_ and the area exposed within one frame A_frame_ is determined using the laser spot radius w_0_, scan speed v, and frame rate f:

(2) $$R_{\text{pixel}\%}=\frac{A_{\text{spot}}}{A_{\text{frame}}}=\;\frac{\pi \;{w_{0}}^{2}}{\pi {w_{0}}^{2}+2w_{0}\frac{\;v}{f}}=\;\frac{1}{1+\frac{2\;v}{\pi \;w_{0}\;f}}$$

This work assumes a circular laser beam with an approximate diameter 2w_0_ of 380 µm along a linear scan vector.

The second metric describes cooling behavior. The decay time t_decay_ and the corresponding thermal signal T_decay_ are determined as the point at which a fitted cooling curve reaches a rate of –1 K/s. This criterion ensures variations in energy input can be reliably detected with minimal standard deviation in automated data processing [16]. To obtain t_decay_ and T_decay_, the raw thermal signals of individual pixels are fitted with a four-parameter hyperbola function:

(3) $$T\left(t\right)=a\;\frac{1}{{\left(t+b\right)}^{c}}+d$$

**Fig. 21:**
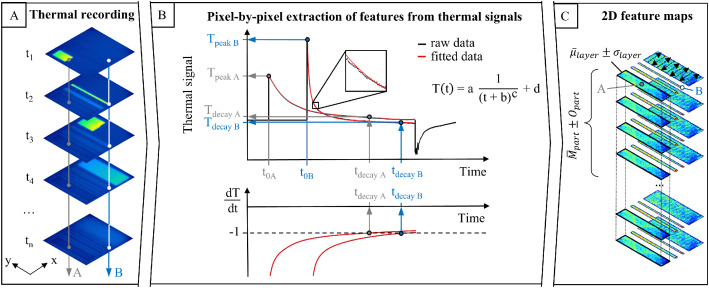
overview of the workflow: exemplary thermograms recorded during processing (A), extraction of T_peak_, t_decay_, and T_decay_ from pixel-level temperature–time profiles (B), and averaging across the component surface and layers (C). Reproduced from [1].

This approach removes noise and short-term fluctuations and enables data reconstruction, significantly compressing file sizes during evaluation. The data processing workflow is schematically visualized in Fig. 21.

Next, the thermal signal of the powder bed surface T_Pre_ was determined 50 frames before laser exposure. Finally, an additional descriptor was introduced to capture the apparent increase in the thermal signal of freshly recoated powder located above previously molten regions. This parameter, denoted ∆T_powder_, is calculated as the temperature difference between powder covering melt regions T_on melt_ and powder without subsurface melt influence T_off melt_:

(4) $$\Delta T_{\text{powder}}=T_{\text{on}\;\text{melt}}-T_{\text{off}\;\text{melt}\;}$$

Evaluation is performed within the same frame taken 0.25 s before laser exposure. This a-posteriori definition minimizes sensitivity to process parameter variations, scan vector lengths, and frame rate, facilitating consistent automated processing. Average values are calculated for all characteristic features of the thermal signals for all exposed pixels per layer, and subsequently over multiple layers, with error propagation applied.

### 4.5 Sample dimensions and density

The thickness of each single layer and the external dimensions of the cuboids were measured using an outside micrometer (accuracy: ± 2 µm) with a built-in torque limiter for consistent measurement force. Each sample is measured at three different positions to ensure reproducible results. Two approaches were applied to determine density:

1. **Volumetric density ρ_Vol_:** The sample mass m_S,a_, was measured on a precision balance (ABJ320, KERN GmbH) and related to the measured length, width, and height:

(5) $$\sigma_{\text{Vol}}=\frac{m_{S,a}}{\text{length}\;\times \;\text{width}\;\times \;\text{height}}$$

2. **Archimedean density ρ_A_:** Following DIN EN ISO 1183–1 (Method A), the sample mass in air m_S,a_ and in ethanol m_S,li_ are recorded. The density of ethanol, used as the immersion medium, was determined with a pycnometer (ρ_li_ = 0.790 g/cm³). The Archimedean density is then calculated as:

(6) $$\sigma_{A}=\frac{m_{S,a}\;\times \;\sigma_{\text{li}}}{m_{S,a}-\;m_{S,\text{li}}}$$

It should be noted that cross-sectional analyses of the samples are not included in this dataset. However, a separate publication [1] discusses cross-sectional images visualizing pore size distribution and edge porosity in the cuboids, melt depth variations in single layers, and track width and depth in single tracks, as well as their potential causes and influence on density and dimensional accuracy.

### 4.6 Tensile testing

Tensile testing (DIN EN ISO 527) of the Type 1A samples (cross-section: 10×4 mm²) is performed on a table-top testing machine (Z2.5, ZwickRoell) using a 2.5 kN load cell at a constant displacement rate of 5 mm/min until failure. The Young's modulus is evaluated between 0.05 and 0.25 % elongation. The stress at break is calculated from the dimensions of each sample, which are measured with a calliper. The displacement of the crosshead is used to determine the elongation at break. All samples are tested in dry conditions. This is achieved by storing the samples in a desiccator for 14 days under ambient pressure with silica gel as a desiccant.

In contrast to the density cuboids, a single contour line is exposed after hatch-filling is complete (post-contour) for the tensile test samples. The scan speed of the contour is equal to the respective hatch-filling parameter. The laser power of the contour is set to 10.8 W for the tensile test samples. In addition, it should be noted that no temperature data were recorded for the tensile test samples, as they are outside the field of view of the thermography camera. Furthermore, the transferability of temperatures recorded for the 10 mm cuboids on tensile test samples is limited because the scan vector length varied between the x- and y-directions in every other layer.

## Limitations

While the dataset provides valuable insights into geometry-dependent thermal signals and their correlation with density and dimensional accuracy in PBF-LB/P, several limitations must be considered when reusing these data. These limitations primarily arise from the physical properties of PA12, the characteristics of infrared thermography, and the specific experimental setup.

• This dataset's IR thermography data does not represent absolute temperatures. The recorded signal depends on emissivity, which varies with material state (powder, molten, solidified) and temperature [9]. Schuffenhauer et al. [17] showed that melting a 200 µm thick layer of PA12 increases transmittance from approx. 18 % (powder) to 45 – 50 % (melt), while absorptivity decreases from about 75 % to 45 % in the wavelength range of CO_2_ lasers (λ = 10.6 µm). However, no optical property data for partially transmissive PA12 are available in the literature for the used photon camera's MWIR range (λ = 1.5 – 5.7 µm). Therefore, the reported thermal data should be regarded as an average over the optical penetration depth, which may span multiple layers rather than a calibrated surface temperature. Accordingly, the thermal features reported here are relative metrics for comparing process conditions and correlating with part properties, not as absolute temperature values.
• The quality of the recorded thermal signals depends on the specifics of the experimental setup, including camera type, process window type, calibration, field of view, and acquisition parameters. Furthermore, the powder bed temperature in this study was controlled at 170 °C, which strongly influences both the baseline signal and the dynamic range of the thermal response. Differences in preheating strategies, recoating methods, or build chamber size in other setups may alter the relative thermal signals. Furthermore, the scan vectors were aligned with the sample length in all layers to isolate directional dimensional deviations. While this design provides controlled insights into geometry-dependent effects, it does not cover alternative scan strategies such as rotated vectors, contour-hatch combinations, or advanced exposure schemes, meaning that these results may not transfer directly to other machines or process conditions without adaptation. Users should therefore exercise caution when generalizing these findings beyond the specific conditions studied. Furthermore, no thermal data was recorded for the tensile test samples since they are positioned outside the field of view of the IR camera.

In summary, the dataset provides robust comparative metrics for process monitoring and quality prediction in PBF-LB/P but is inherently limited by emissivity and transmissivity uncertainties, setup dependence, and material specificity. The recorded signals do not reflect absolute surface temperatures and cannot resolve phase-transition temperatures with sufficient accuracy to benchmark heat transfer or crystallization models. Instead, the dataset is best suited for identifying correlations between thermal signal features and part properties, benchmarking monitoring strategies, or training data-driven models for quality prediction and process control.

## Ethics Statement

The authors have read and follow the ethical requirements for publication in Data in Brief and confirm that the current work does not involve human subjects, animal experiments, or any data collected from social media platforms.

## CRediT Author Statement

**Joseph Hofmann:** Conceptualization, Methodology, Software, Validation, Formal analysis, Investigation, Data curation, Visualization, Writing – original draft, Writing – review & editing, Supervision, Project administration. **Benedikt Burchard:** Validation. **Valentin Krieger:** Software, Formal analysis, Investigation, Data curation. **Katrin Wudy:** Conceptualization, Resources, Writing – review & editing, Supervision, Project administration

## Acknowledgements

This research did not receive any specific grant from funding agencies in the public, commercial, or not-for-profit sectors.

## Declaration of Competing Interest

The authors declare that they have no known competing financial interests or personal relationships that could have appeared to influence the work reported in this work.

## Data Availability

MediaTUMDataset for Analysis of Geometry-dependent Temperature-Time-Profiles in Laser-based Powder Bed Fusion of Polyamide 12 (Original data).

## References

[1] Hofmann J., Burchard B., Krieger V., Wudy K. (2025). Influence of geometry-dependent temperature-time-profiles on density and dimensional accuracy in laser-based powder bed fusion of polyamide 12. Mater. Des..

[2] D. Tasch, A. Mad, R. Stadlbauer, M. Schagerl, Thickness dependency of mechanical properties of laser-sintered polyamide lightweight structures, 2214-8604 23 (2018) 25–33. 10.1016/j.addma.2018.06.018.

[3] Gelaziene E., Milasiene D. (2023). Influence of the type of plastic and printing technologies on the compressive behavior of 3D-printed heel prototypes. Mater. (Basel).

[4] Tikhomirov E., Åhlén M., Di Gallo N., Strømme M., Kipping T., Quodbach J., Lindh J. (2023). Selective laser sintering additive manufacturing of dosage forms: effect of powder formulation and process parameters on the physical properties of printed tablets. Int. J. Pharm..

[5] Greiner S. (2023).

[6] J. Hofmann, Z. Li, K. Taphorn, J. Herzen, K. Wudy, Porosity prediction in laser-based powder bed fusion of polyamide 12 using infrared thermography and machine learning, 2214-8604 85 (2024) 104176. 10.1016/j.addma.2024.104176.

[7] Greiner S., Jaksch A., Cholewa S., Drummer D. (2021). Development of material-adapted processing strategies for laser sintering of polyamide 12. Adv. Ind. Eng. Polym. Res..

[8] Reinhardt T. (2016).

[9] A. Sommereyns, S. Leupold, F. Rudlof, M. Willeke, A. Ghosh, B. Ohannessian, D. Merwin, R. Elahi, S. Barcikowski, N. Vogel, M. Schmidt, Light matters: quantifiable optical interaction between near-infrared laser radiation and nano-additivated polymers in all states of matter present in laser powder bed fusion, 2214-8604 86 (2024) 104211. 10.1016/j.addma.2024.104211.

[10] Chatham C.A., Bortner M.J., Johnson B.N., Long T.E., Williams C.B. (2021). Predicting mechanical property plateau in laser polymer powder bed fusion additive manufacturing via the critical coalescence ratio. Mater. Des..

[11] Wroe W.W., Gladstone J., Phillips T., Fish S., Beaman J., McElroy A. (2016). In-situ thermal image correlation with mechanical properties of nylon-12 in SLS. RPJ.

[12] F. Sillani, E. MacDonald, J. Villela, M. Schmid, K. Wegener, In-situ monitoring of powder bed fusion of polymers using laser profilometry, 2214-8604 59 (2022) 103074. 10.1016/j.addma.2022.103074.

[13] Southon N., Stavroulakis P., Goodridge R., Leach R. (2018). In-process measurement and monitoring of a polymer laser sintering powder bed with fringe projection. Mater. Des..

[14] Klamert V., Schiefermair L., Bublin M., Otto A. (2023). Situ analysis of curling defects in powder bed fusion of polyamide by simultaneous application of laser profilometry and thermal imaging. Appl. Sci..

[15] Hofmann J., Burchard B., Krieger V., Wudy K. (2025). Dataset for analysis geometry-dependent temperature-time-profiles in laser-based powder bed fusion of polyamide 12. Tech. Univ. Munich MediaT..

[16] Hofmann J., Wudy K. (2022). In situ process monitoring in laser-based powder bed fusion of polyamide 12 using thermal imaging. Int. J. Adv. Manuf. Technol..

[17] Schuffenhauer T., Stichel T., Schmidt M. (2021). Employment of an extended double-integrating-sphere system to investigate thermo-optical material properties for powder bed fusion. J. Mater. Eng. Perform..

